# Effects of metformin on acute respiratory distress syndrome in preclinical studies: a systematic review and meta-analysis

**DOI:** 10.3389/fphar.2023.1215307

**Published:** 2023-09-28

**Authors:** Liu Wang, Yan-Fen Tian, Wen-Qing Deng

**Affiliations:** ^1^ Department of Respiratory and Critical Care Medicine, ChangChun Central Hospital, Changchun, Jilin, China; ^2^ Department of Ophthalmology, Changchun Aier Eye Hospital, Changchun, Jilin, China; ^3^ Ophthalmology Department of Putuo District People’s Hospital of Zhoushan City, Zhoushan, Zhejiang, China

**Keywords:** critical illness, lung injury, respiratory distress syndrome, mortality, inflammation

## Abstract

**Introduction:** In this study, we conducted a systematic review and meta-analysis to judge the effects of metformin on acute respiratory distress syndrome (ARDS) in a comprehensive and quantitative manner.

**Methods:** We included studies that tested the effects of metformin on ALI or ARDS in *in vivo* studies. We excluded literature from which data could not be extracted or obtained. Electronic search was conducted to retrieve relevant literature from public databases, including PubMed, Web of Science, Embase, Scopus, and the Cochrane Central Register of Controlled Trials (inception to July 2023). Moreover, ProQuest Dissertations and Theses Global, Google Scholar, and Baidu scholar were inquired. Retrieved literature was screened and evaluated by pairs of reviewers independently according to pre-stated criteria. The Systematic Review Center for Laboratory Animal Experimentation risk of bias tool was used to evaluate the methodological quality of eligible literature. No restriction was exerted on publication status or language.

**Results:** Fifteen preclinical studies were analyzed in this meta-analysis. Pooled results showed metformin effectively decreased pulmonary wet-to-dry weight ratios [SMD = −2.67 (−3.53 to −1.81), I^2^ = 56.6%], protein content [SMD = −3.74 (−6.76 to −0.72), I^2^ = 86.7%] and neutrophils [SMD = −3.47 (−4.69 to −2.26), I^2^ = 0%] in BALF, pulmonary malondialdehyde [SMD = −1.98 (−3.77 to −0.20), I^2^ = 74.2%] and myeloperoxidase activity [SMD = −3.15 (−4.79 to −1.52), I^2^ = 74.5%], lung injury scores [SMD = −4.19 (−5.65 to −2.74), I^2^ = 69.1%], and mortality at 24 h [RR = 0.43 (0.24–0.76), I^2^ = 0%] as well as 48 and 72 h.

**Conclusion:** Metformin inhibited pulmonary inflammation and oxidative stress and improved experimental lung injury and survival rates in animal models of ARDS. Results from randomized controlled trials are needed.

## Introduction

As the global pandemic of COVID-19 continues, cases of COVID-19-associated acute respiratory distress syndrome (ARDS) are overwhelming many clinical centers in epidemic areas (Meyer et al., 2021). As a common type of critical illness, ARDS still has an unacceptable high mortality, possibly due to its complexity of etiology and pathophysiological mechanisms (Meyer et al., 2021). Nevertheless, the endeavor quest for effective therapeutic strategies has never been hindered. In addition to diverse mechanical ventilation maneuver and extracorporeal membrane oxygen, finding novel and efficacious pharmacological agents for ARDS is a research highlight (Meyer et al., 2021; Tao et al., 2022).

Metformin, dimethyl biguanide, is known as an anti-diabetic agent for several decades (Triggle et al., 2022). Hitherto, it is the first-choice antihyperglycemic agent for many patients with diabetes. Diabetes, especially, diabetes mellitus type 2, is a common disease in individuals (Zheng et al., 2018). The morbidity of diabetes mellitus type 2 is approximately 8% in adults (Zheng et al., 2018). In consequence, preexisted diabetes in critically ill patients is common. A recent meta-analysis showed that reduced mortality was associated with metformin use in COVID-19 patients with diabetes (Poly et al., 2021). Recent evidence also indicated that metformin may be protective for diabetic patients with critical illness. Evidence showed that preoperative metformin exposure in adult patients with diabetes mellitus type 2 was associated with decreased risk-adjusted mortality as well as readmission after a major operation (Reitz et al., 2020). In patients with type 2 diabetes who were admitted to the intensive care unit, 30-day mortality decreased in preadmission metformin users (Christiansen et al., 2013). Moreover, a meta-analysis involving 11 trials suggested the benefit of preadmission metformin use on the reduction in mortality in septic patients with diabetes mellitus (Li et al., 2021). A recent report showed that in patients with type 2 diabetes mellitus and sepsis, 90-day mortality was reduced in users of metformin who used it during hospitalization (Gómez et al., 2022).

ARDS is a rational research focus for critical illness, due its high morbidity. Sepsis is known as one of the most common risk factors for ARDS (Meyer et al., 2021). As metformin has shown its role in reducing mortality in septic patients (Li et al., 2021; Gómez et al., 2022), it is possible that metformin may also benefit ARDS. A number of preclinical studies have highlighted the effect of metformin on acute lung injury (ALI), a mild form of ARDS (Wu et al., 2019; Medeiros et al., 2021; Xian et al., 2021; Yuan et al., 2022). Reduced mortality, pulmonary wet-to-dry weight ratio (W/D), lung injury scores, protein in bronchoalveolar lavage fluid, and levels of malondialdehyde (MDA) as well as myeloperoxidase (MPO) in the lungs were announced in those studies. Moreover, in a diabetic animal model, dampened ventilator-induced lung injury and gas exchange impairment by metformin were reported (Schranc et al., 2022). These results prompt us to conduct a meta-analysis to probe the effect of metformin on ARDS in a comprehensive and quantitative manner, which may be a promising strategy for repurposing metformin for the improvement of ARDS. This meta-analysis aimed to explore the efficacy of metformin as an intervention for ARDS in preclinical studies or randomized controlled trials. The primary outcome was lung injury evidence. The second objective was mortality.

## Methods

To report this systematic review scientifically and rigorously, we followed the guidelines of the Preferred Reporting Items for Systematic Reviews and Meta-Analyses (PRISMA). No restriction was exerted on publication status or language.

### Inclusion criteria

We included studies that tested the effects of metformin on ALI or ARDS in *in vivo* studies, irrespective of whether there was a clinical trial or not: 1) preclinical experiments or randomized controlled trials; 2) animal models preclinically compatible with ARDS or patients with ARDS; 3) prophylactic or therapeutic administration of metformin as the intervention; 4) routine treatment or placebo as the control; and 5) the primary outcome was indicators of ARDS such as W/D and lung injury scores. The second outcome was mortality.

### Exclusion criteria

The exclusion criteria are as follows: 1) Observational studies; 2) duplicated publication; 3) *in vitro* or *ex vivo* study; 4) metformin combined with other medicines; 5) compared metformin with other medicines; 6) evidence of lung injury was obtained 7 days after induction in preclinical experiments; 7) evidence of lung injury could not be found in the literature; and 8) data could not be extracted or obtained even after contacting the original authors by email.

### Search strategies

From their inception to July 2023, databases including PubMed, Web of Science, Embase, Scopus, and the Cochrane Central Register of Controlled Trials were inquired to retrieve the relevant literature by using search terms as follows: 1) metformin, dimethylbiguanidine, dimethylguanylguanidine, and glucophage; 2) acute lung injury, ALI, ARDS, acute respiratory distress syndrome, and respiratory distress syndrome (Supplementary Table S1). Moreover, ProQuest Dissertations and Theses Global, Google scholar, and Baidu Scholar were also inquired. We also examined reference lists of reviews and potential eligible studies to find relative studies.

### Study selection

The retrieved literature was screened using endnote software (Clarivate Analytics, United States) first to delete duplicates. Then, titles and abstracts of remains were screened by pairs of reviewers. After that, the full texts of potential eligible studies were reviewed and judged. If any conflicts exist, it would be adjudicated by a third reviewer. Reference lists were also examined to find the potential eligible literature.

ALI in animals was defined by using authors’ definitions or evidence of lung injury, such as lung histopathological analysis, protein content or neutrophils in BALF, and W/D, provided within 7 days after ALI induction. ARDS was defined by using Berlin definition (Ranieri et al., 2012).

### Data extraction

To evaluate the methodological quality of the eligible literature, the Systematic Review Center for Laboratory Animal Experimentation (SYRCLE) risk of bias tool (Hooijmans et al., 2014) or Cochrane’s “Risk of bias” tool (Higgins et al., 2011) was used for preclinical studies or randomized controlled trials, respectively.

We adopted a standardized form for data extraction. The following items were extracted by two reviewers independently: characteristics of studies including the first author’s names and countries, the publication year, animals, ALI models, and strategies of metformin administration. The primary outcome was lung injury indicators such as lung injury scores, W/D, indicators of pulmonary edema or neutrophil infiltration in the lung, and an index of oxidative stress. The secondary objective was mortality. The primary and secondary objectives were evaluated as pulmonary edema, inflammation, and destruction of lung tissues are the evidence of lung injuries, and mortalities are the clinical concerns.

The standard error of the mean value was converted to the standard deviation value. The most effective result was pooled, if results from multiple time points or various doses intervention were provided in one study.

### Statistical analysis

We performed meta-analysis using STATA SE 14.0 statistical software for Windows (StataCorp LP, TX, United States). Homogeneity between studies was judged using Cochran’s Q statistic and *I* ^*2*^ statistic. It denotes a significant or high heterogeneity, if *p* ≤ 0.10 (Cochran’s Q statistic) or *I* ^*2*^ statistic ≥50%, respectively. A random-effects model will be used if significant or high heterogeneity was identified. Otherwise, a fixed-effects model was used. We also performed subgroup analysis to investigate potential heterogeneity.

## Results

**FIGURE 1:**
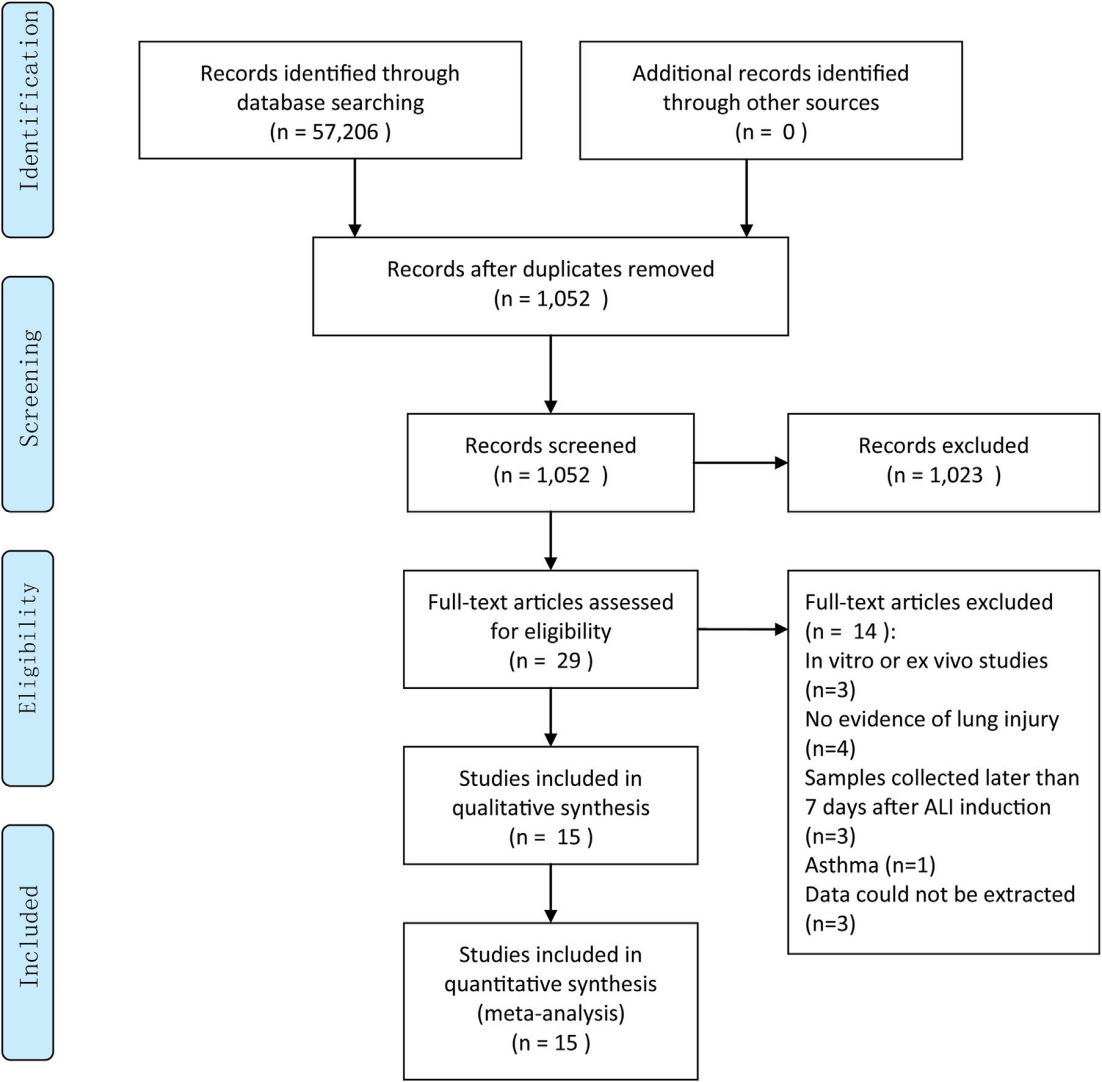
Literature selection.

Initially, we retrieved a total of 57, 206 pieces of literature from electronic search. Then, we screened, reviewed, and excluded most of these articles as not meeting our criteria stated in the method section. Finally, we included 15 preclinical studies (Zmijewski et al., 2008; Jian et al., 2013; Liu et al., 2016; Vaez et al., 2016; Wang et al., 2016; Ghavimi et al., 2018; Wu et al., 2018; Yu et al., 2018; He et al., 2019; Wu et al., 2019; Xian et al., 2021; Zhang Y. et al., 2022; Zhang M. et al., 2022; Yuan et al., 2022; Wan et al., 2023) in this meta-analysis. The procedure for study selection is presented in a flow chart in Figure 1.

### Study characteristics

We present the characteristics of 15 preclinical studies in Table 1. As shown in Table 1, the preclinical studies were published between 2008 and 2023. Healthy mice or rats were used to establish ALI models, which were induced by using lipopolysaccharide (LPS), cecal ligation and puncture, paraquat, or gas explosion. Almost half of the studies used rats including Sprague Dawley rats and Wistar rats to establish animal models. In addition, three types of mice were used in other studies. Either prophylactic or therapeutic administration of metformin was investigated in 14 studies. One study reported both the prophylactic and therapeutic administration of metformin. Results from the therapeutic administration of metformin were reported in seven studies. Metformin was administrated via intraperitoneal injection in most studies. Oral administration of metformin was investigated in three studies. Lung injuries were evaluated from 8 h to 7 days after induction. Nine studies reported lung injury indicators at less than or equal to 24 h. Two studies reported indicators of lung injuries at 7 days.

**TABLE 1:** Characteristics of included preclinical studies.

Author/year	Country	Animal	ALI model	Metformin administration
Zmijewski/2008	United States	C57BL/six mice	LPS it	Metformin (125 mg/kg i.p.) given 0.5 and 8 h after LPS administration
Jian/2013	United States	SD rats	LPS ip	Metformin (15 mg/kg) i.p. 6 h after LPS treatment
Wang/2016	China	BALB/c mice	LPS it	Metformin (250 mg/kg) i.p. 0.5 h prior to LPS administration
Vaez/2016	Iran	Wistar rats	LPS i.p.	Metformin (100 mg/kg, i.p.) 30 min before LPS injection
Liu/2016	United States	C57BL/six mice	CLP, hemorrhage + CLP	Metformin (100 mg/kg) i.p. 48 h, 24 h, and 30 min prior to CLP. For combination of hemorrhage and CLP experiment, the second dose of metformin was given before hemorrhage
Ghavimi/2018	Iran	Wistar rats	CLP	Metformin (50 or 100 mg/kg) i.p. 2 h after CLP
Wu/2018	China	BALB/c mice	LPS i.p.	Metformin (400 mg/kg) i.p. 30 min before LPS
Yu/2018	China	BALB/c mice	LPS i.p.	Metformin (50 mg/kg) orally administrated for 7 days
He/2019	China	C57BL/six mice	LPS it	Metformin (100 mg/kg) i.p. 30 min before LPS
Wu/2019	China	SD rats	Paraquat i.p.	Metformin (400 mg/kg) intragastric administration 2 h after paraquat i.p. All rats received an intragastric treatment once a day for 7 days at a fixed time every morning
Xian/2021	United States	C57BL/six mice	LPS i.p.	Metformin (10 or 50 mg/kg) i.p. for 3 consecutive days with the last dose administered 30 min before LPS administration or metformin (400 mg/kg) in the drinking water *ad libitum* starting 2 days prior to LPS challenge or metformin (50 mg/kg) i.p. 30 min after LPS injection with a second dose given after 24 h
Zhang M/2022	China	SD rats	Blast lung injury	Metformin 10 mg/kg i.p. daily after the explosion
Zhang Y/2022	China	ICR mice	LPS it.	Metformin 50 mg/kg i.p. 30 min prior to the LPS challenge
Yuan/2022	China	SD rats	Paraquat i.p.	Metformin 35 mg/kg i.p. 1 h after paraquat administration followed by metformin 35 mg/kg i.p. once a day

CLP, cecal ligation and puncture; ICR, Institute of Cancer Research; i.p.,intraperitoneal injection; it., intratracheal injection; LPS, lipopolysaccharide; SD, Sprague Dawley.

### Risk of bias assessment

The risk of bias from preclinical studies was assessed using SYRCLE’s risk of bias tool. Detailed information is shown in appendix Supplementary Table S1.

### Lung injury

**FIGURE 2:**
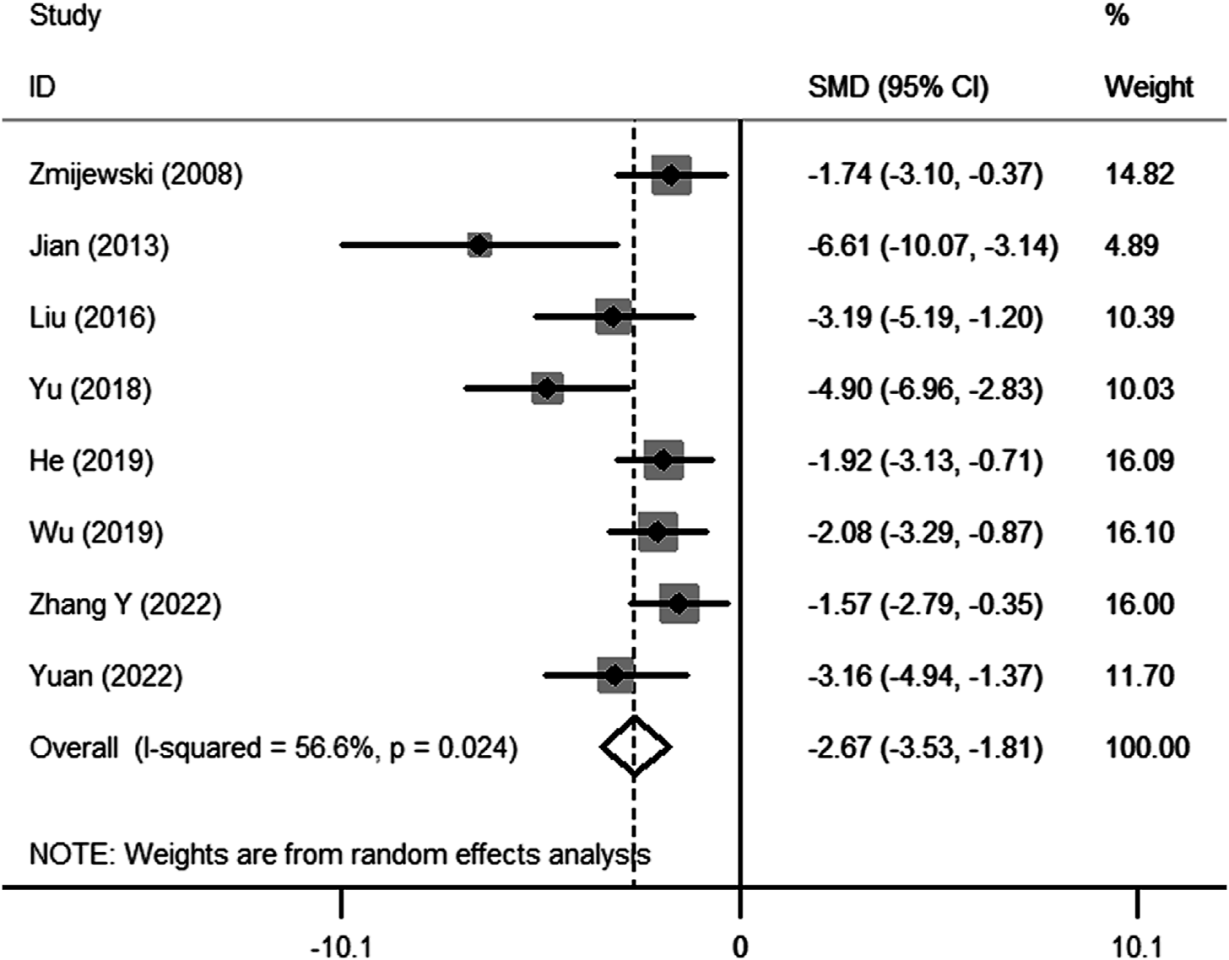
Forest plots for pulmonary wet-to-dry weight ratio in preclinical studies.

**FIGURE 3:**
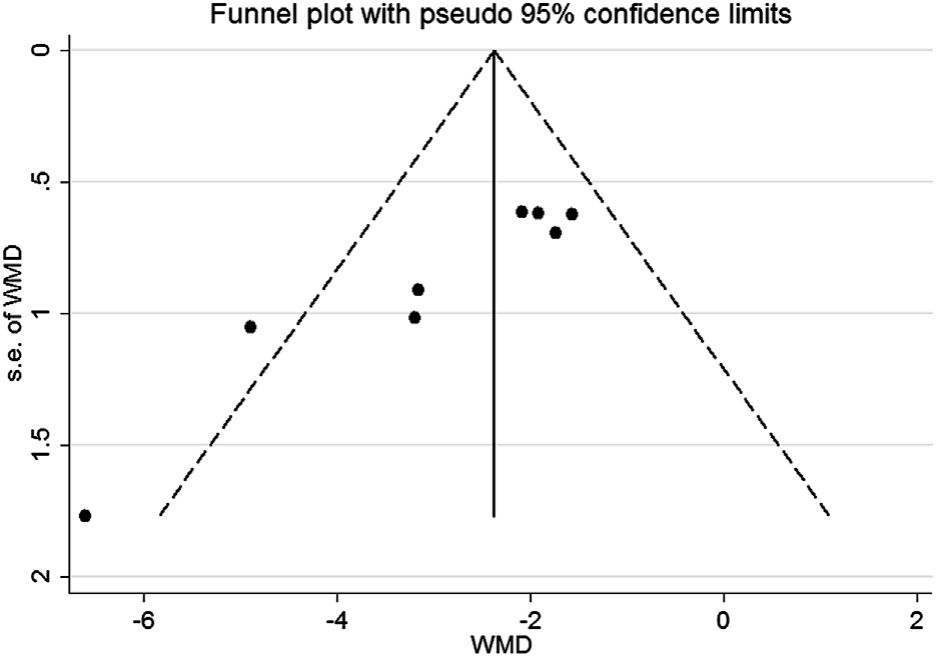
Funnel plot.

**FIGURE 4:**
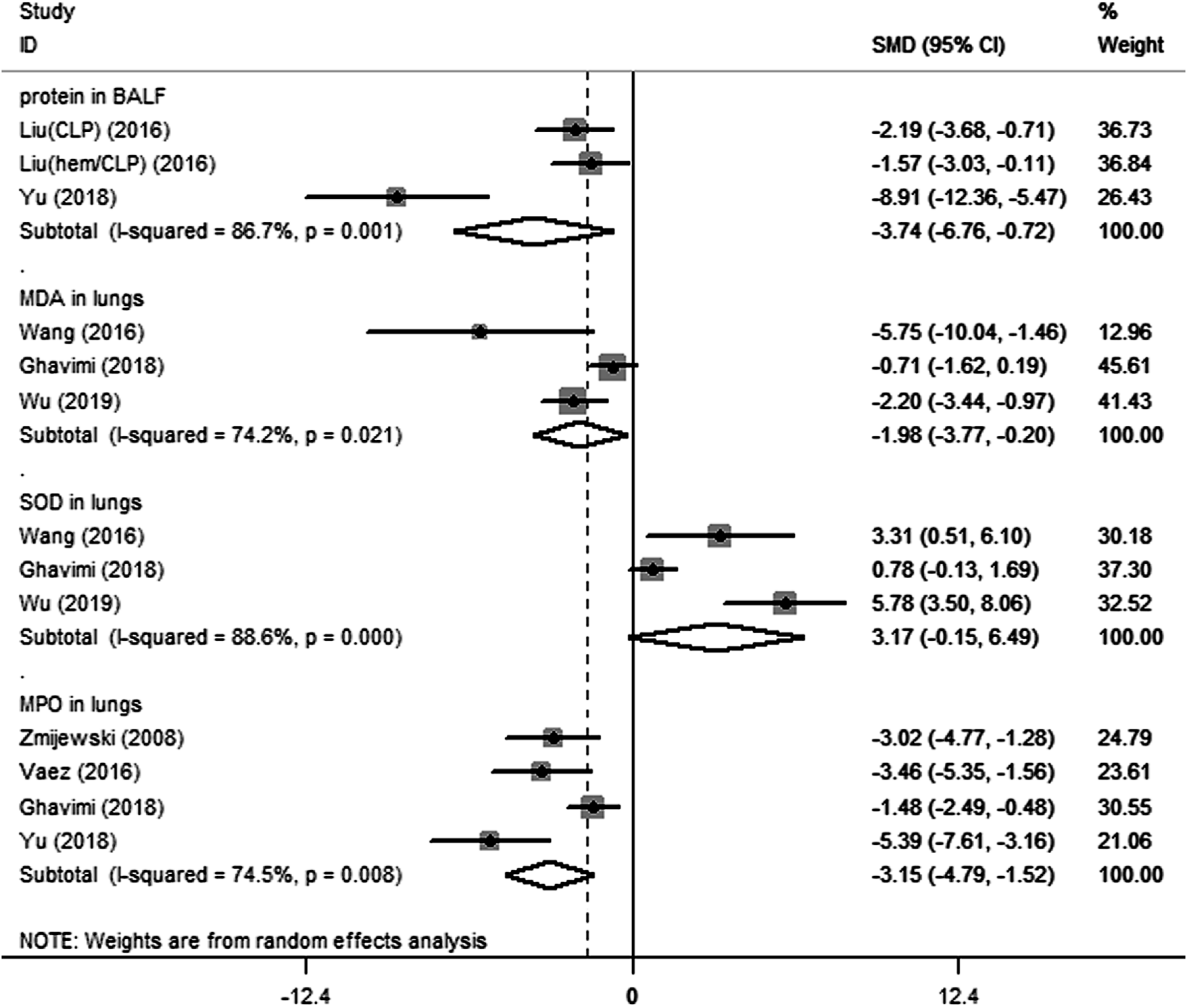
Forest plots for protein in bronchoalveolar lavage fluid, and malondialdehyde, superoxide dismutase, and myeloperoxidase in lung in preclinical studies.

**FIGURE 5:**
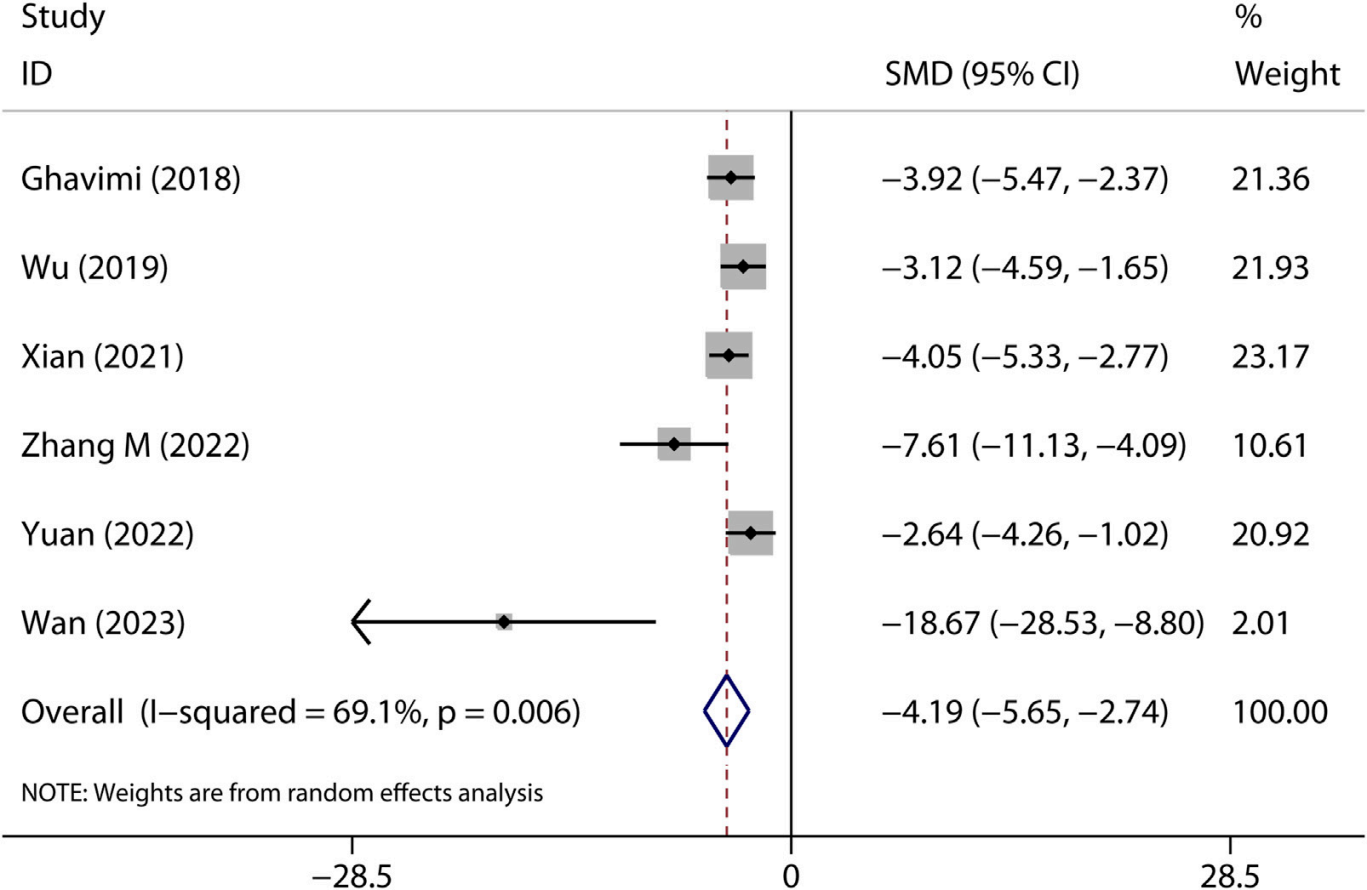
Forest plots for lung injury scores in preclinical studies.

Fifteen preclinical studies reported the effects of metformin on experimental ARDS. Lung injury was evaluated with the indicators of lung edema, inflammatory cell infiltration, oxidative stress, and lung histopathological analysis. In general, a declining value of indicators suggests an improved result, in addition to the value of anti-oxidative enzymes. W/D is a sensitive indicator for the measurement of lung edema. The W/D was reported in eight studies. Metformin effectively decreased W/D [SMD = −2.67 (−3.53 to −1.81); Figure 2]. Potent publication bias was investigated using the funnel plot (Figure 3). Protein content in BALF could be used to evaluate endothelial permeability. The protein content in BALF was reported in two studies. He et al. (2019) reported the results of protein content in BALF using two different types of animal models, a total of three results were pooled. The pooled result suggests that metformin dampened protein content in BALF [SMD = −3.74 (−6.76 to −0.72); Figure 4]. MDA is an oxidative stress indicator, while superoxide dismutase (SOD) is an important anti-oxidative stress substance. Three studies reported both MDA and SOD. As shown in Figure 4, pooled results showed that metformin reduced MDA [SMD = −1.98 (−3.77 to −0.20)] but did not elevate SOD [SMD = 3.17 (−0.15–6.49)]. MPO is an enzyme that is mainly generated in neutrophils. The activity of MPO is used as an indicator of neutrophil activation and infiltration. The pooled result of MPO including four studies showed reduced MPO activity in metformin-treated animals [SMD = −3.15 (−4.79 to −1.52); Figure 4]. In parallel, counts of neutrophils in BALF were also decreased in metformin-treated animals [SMD = −3.47 (−4.69 to −2.26) and I^2^ = 0%]. The lung injury score is a quantified strategy to investigate the extent of lung injury based on pulmonary tissue sections. Six studies reported lung injury scores in animals that received metformin versus vehicle [SMD = −4.19 (−5.65 to −2.74) and I^2^ = 69.1%; Figure 5].

### Mortality

**FIGURE 6:**
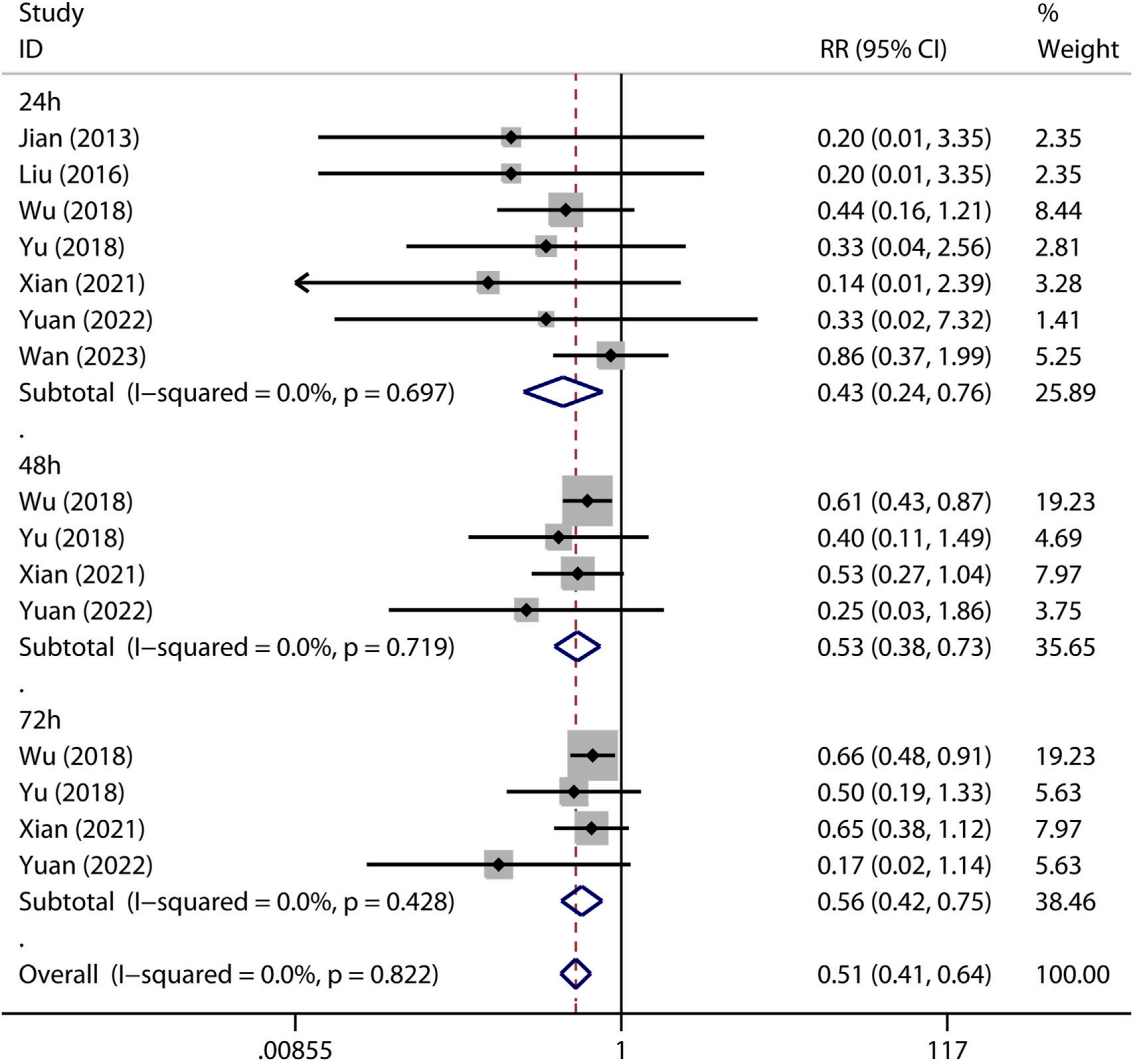
Forest plots for mortality in preclinical studies.

Mortality is a major concern of ARDS treatment. The effect of metformin on mortality was reported in experimental ARDS animal models. Seven studies reported mortality at 24 h after the induction of experimental ARDS in animals that received metformin versus vehicle [RR = 0.32 (0.15–0.68); Figure 6]. Moreover, improved mortality by metformin at 48 and 72 h was reported in four studies (Figure 6).

### Subgroup analysis

#### W/D

Metformin reduced W/D in both direct [three studies, SMD = −1.74 (−2.47 to −1.02), I^2^ = 0%] and indirect [five studies, SMD = −3.57 (−4.91 to −2.23), I^2^ = 58.2%] ALI. Metformin pre-treatment [four studies, SMD = −2.68 (−4.00 to −1.36), I^2^ = 65.1%] or post-treatment [four studies, SMD = −2.76 (−4.12 to −1.40), I^2^ = 60.1%] reduced W/D. Moreover, either a low dose [four studies, SMD = −3.70 (−5.69 to −1.70), I^2^ = 76.2%] or high dose [four studies, SMD = −2.07 (−2.76 to −1.39), I^2^ = 0%] of metformin significantly reduced W/D.

#### Lung injury scores

Effects of metformin on direct ALI were reported in one study (Zhang M. et al., 2022) [SMD = −7.61 (−11.13 to −4.09)]. Indirect ALI was also reduced by metformin [five studies, SMD = 3.72 (−5.07 to −2.37), I^2^ = 64.8%]. Similarly, only one study (Xian et al., 2021) reported pre-treatment of metformin on the lung injury scores [SMD = −4.05 (−5.33 to −2.77)]. Post-treatment of metformin reduced the lung injury scores [five studies, SMD = −4.49 (−6.48 to −2.49), I^2^ = 74.8%]. Ghavimi et al. (2018) reported that both low (50 mg/kg) and high (100 mg/kg) doses of metformin have a similar effect on lung injury scores. We pooled it in the high-dose group. Either low [three studies, SMD = −4.22 (−6.22 to −2.21), I^2^ = 69.8%] or high [three studies, SMD = −4.67 (−7.62 to −1.72), I^2^ = 79.0%] doses of metformin reduced the lung injury scores.

#### Mortality

**FIGURE 7:**
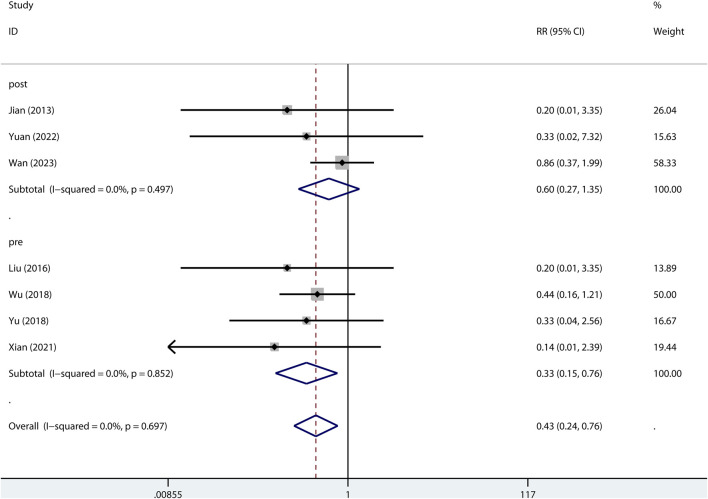
Forest plots for the effect of post- or pre-treatment of metformin on mortality at 24 h in preclinical studies.

**FIGURE 8:**
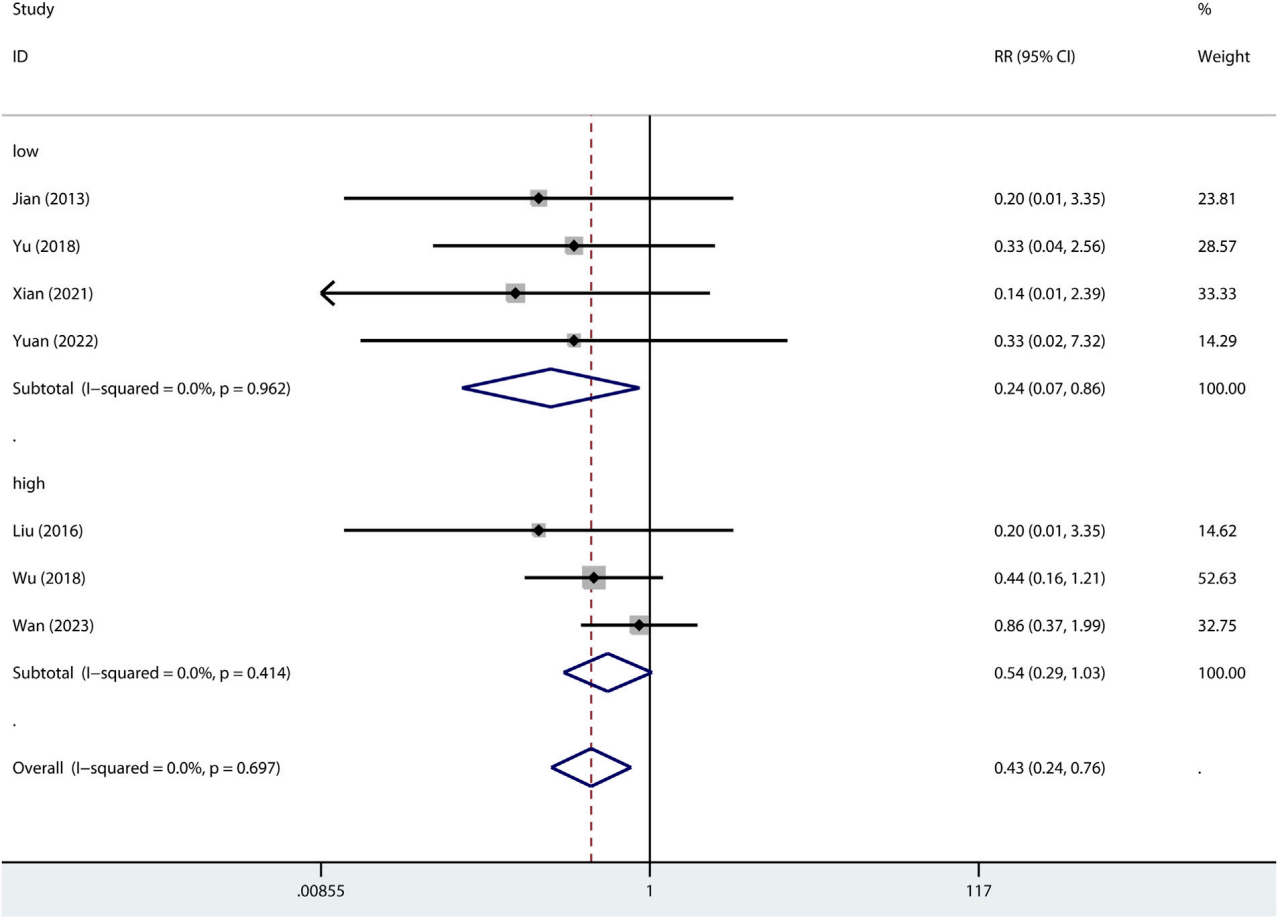
Forest plots for the effect of low or high dose of metformin on mortality at 24 h in preclinical studies.

The 24-h mortality was reported in indirect ALI only. As shown in Figure 7, pre-treatment but not post-treatment of metformin reduced the 24-h mortality. Interestingly, a low dose but not high dose of metformin reduced 24-h mortality (Figure 8).

## Discussion

Metformin has pleiotropic properties (Triggle et al., 2022). Interests in repurposing metformin are rapidly growing (Triggle et al., 2022). Our meta-analysis based on the pooled results of preclinical studies showed that metformin reduced pulmonary edema and inflammation and increased survival rate. However, evidence from clinical studies is still needed to verify the animal study results.

### Mechanisms of metformin on lung injury

It is interesting that a traditional anti-diabetic agent can reduce lung injury. How did metformin do it? Several studies have shed light on it.

### Anti-edematogenic effects

Refractory hypoxemia is a feature of ARDS. Increased endothelial permeability due to damage of alveolar capillaries contributes to substantial edema fluid gathering in the alveolar space and pulmonary interstitium that causes impairment of gas exchange (Meyer et al., 2021). Activation of AMP-activated protein kinase (AMPK)-1 isoforms dampened the deterioration of endothelial permeability triggered by LPS (Jian et al., 2013). Metformin can activate AMPK-1 isoforms and reverse the LPS-induced decrease in the pulmonary microvascular endothelial cell barrier resistance and impairment of wound repair in the endothelium that resulted in the improvement of endothelial permeability and pulmonary edema (Jian et al., 2013). Moreover, enhanced transendothelial resistance of endothelial monolayers by metformin was reported in an *in vitro* study (Uddin et al., 2021). In an *ex vivo* study, the high-pressure ventilation-induced increase in microvascular permeability and edema formation in rabbit lung was notably improved in the metformin-pretreated group (Tsaknis et al., 2012).

### Anti-inflammatory effects

Inflammasome activation contributes to inflammation. Metformin inhibited LPS-triggered activation of the NLRC4 inflammasome by upregulating AMPK phosphorylation (He et al., 2019). Activation of AMPK by metformin may also trigger mitophagy-mediated inhibition of NLRP3 inflammasome activation, as metformin is capable of induction of mitophagy (Boyle et al., 2018). Moreover, the LPS-induced increase in cleaved caspase-1, IL-1β production, and the N-terminal fragment of gasdermin D was reduced in metformin-treated animals (Zhang Y. et al., 2022). The effect of metformin on pyroptosis was associated with elevating sirtuin-1 expression and inhibiting NLRP3 inflammasome activation (Zhang Y. et al., 2022). In LPS-challenged macrophages, the inhibiting effects of metformin on NLRP3 inflammasome activation and pyroptosis were also detected (Xian et al., 2021). However, the inhibiting effect of metformin on NLRP3 inflammasome activation in LPS-challenged macrophages was not AMPK-dependent but ATP-dependent mitochondrial DNA synthesis (Xian et al., 2021). Mitochondrial DNA is the ligand of NLRP3. The conjunction of mitochondrial DNA and NLRP3 resulted in NLRP3 inflammasome activation (Zhong et al., 2018). As metformin inhibits ATP production (Wheaton et al., 2014), a decrease in the ATP-dependent mitochondrial DNA production was detected in metformin-treated and LPS-primed macrophages (Xian et al., 2021).

NF-κB triggers a key role in inflammation such as the regulation of cytokine production. LPS-stimulated NF-κB p65 subunit phosphorylation, degradation of IκB-α, and accumulation of NF-κB in the nucleus of neutrophils or alveolar macrophages could be inhibited by metformin via inhibiting mitochondrial respiratory complex I activation (Zmijewski et al., 2008).

Moreover, in the present meta-analysis, although pooled results showed that metformin did not increase SOD levels; the SOD level was elevated in two of the three trials included. As an anti-oxidative stress substance, SOD plays an important role in protecting the lung. In addition to having anti-oxidative effect within cells, extracellular superoxide dismutase in alveolar fluid is protective for methicillin-resistant *Staphylococcus aureus* inoculation-triggered inflammation and acute lung injury (Sul et al., 2023).

Collectively, anti-inflammatory and anti-edematogenic effects of metformin favor the amelioration of lung injury.

### Mortality and weaknesses

Improved mortality at 24–72 h was detected in pooled preclinical studies. Further subgroup analysis showed that only pre-treatment of metformin has benefits on 24-h mortality in indirect ALI. However, post-treatment has more clinical practice meanings. To our surprise, a high dose of metformin (≥100 mg/kg) did not improve 24 h mortality in indirect ALI; on the contrary, a low dose of metformin (<100 mg/kg) significantly reduced 24-h mortality. Its potential mechanisms are still needed to be elucidated. These preclinical studies only reported mortality during a short period. Two retrospective clinical studies reported that all-cause mortality at 30 or 90 days was not improved in preadmission metformin users compared with metformin non-users in diabetic patients with ARDS (Jo et al., 2017; Oh and Song, 2020). Nevertheless, there are several limitations in the two clinical studies. First, all the two clinical studies were observational and conducted in the same country, Korea. Importantly, it is unclear that whether the preadmission metformin users continued using metformin during ARDS. Commonly, oral anti-diabetic agents will be substituted with insulin when diabetic patients suffer a critical illness. Accordingly, both the previous metformin users and non-users will use insulin, giving the result no meaning.

In addition to its anti-diabetic use, metformin has shown its effects in treating a variety of disorders such as cancer, polycystic ovary syndrome, and COVID-19 (Triggle et al., 2022). For critical illness, metformin has been suggested as a potent therapeutic intervention for patients with severe traumatic brain injury (Taheri et al., 2019). Improved mortality was reported in septic patients with diabetes mellitus that were treated with metformin (Li et al., 2021; Gómez et al., 2022). This evidence suggests the potential use of metformin for critically ill patients. Nevertheless, well-designed randomized controlled trials are still needed to assess the effects of metformin on ARDS in diabetic or non-diabetic patients.

## Conclusion

Evidence from this meta-analysis suggests the possibility of metformin for the treatment of ARDS. Metformin inhibits pulmonary inflammation and oxidative stress and improves experimental lung injury and survival rates in animal models of ARDS. Results from randomized controlled trials are needed.

## Data Availability

The original contributions presented in the study are included in the article/Supplementary Material; further inquiries can be directed to the corresponding author.
