# Supplementary material for: Effects of metformin on acute respiratory distress syndrome in preclinical studies: a systematic review and meta-analysis
Source: Front Pharmacol. 2023 Sep 28;14:1215307. doi: 10.3389/fphar.2023.1215307 (PMC10568015; doi:10.3389/fphar.2023.1215307)
Supplement: Supplementary file 1 [file Table1.doc]

**Table S1. Risks of bias evaluated by the SYRCLE’s risk of bias tool.**

| **Author**  **/Year** | 1 | 2 | 3 | 4 | 5 | 6 | 7 | 8 | 9 | 10 |
| --- | --- | --- | --- | --- | --- | --- | --- | --- | --- | --- |
| Zmijewski  /2008 | Unclear | Unclear | Unclear | Yes | Unclear | Unclear | Unclear | Yes | Yes | Yes |
| Jian/2013 | Unclear | Unclear | Unclear | Yes | Unclear | Unclear | Unclear | Yes | Yes | Yes |
| Wang  /2016 | Unclear | Unclear | Unclear | Yes | Unclear | Unclear | Unclear | Yes | Yes | Yes |
| Vaez/2016 | Unclear | Unclear | Unclear | Yes | Unclear | Unclear | Unclear | Yes | Yes | Yes |
| Liu/2016 | Unclear | Unclear | Unclear | Yes | Unclear | Unclear | Unclear | Yes | Yes | Yes |
| Ghavimi  /2018 | Unclear | Unclear | Unclear | Yes | Unclear | Unclear | Unclear | Yes | Yes | Yes |
| Wu/2018 | Unclear | Unclear | Unclear | Yes | Unclear | Unclear | Unclear | Yes | Yes | Yes |
| Yu/2018 | Unclear | Unclear | Unclear | Yes | Unclear | Unclear | Unclear | Yes | Yes | Yes |
| He/2019 | Unclear | Unclear | Unclear | Yes | Unclear | Unclear | Unclear | Yes | Yes | Yes |
| Wu/2019 | Yes | Unclear | Unclear | Yes | Unclear | Unclear | Unclear | Yes | Yes | Yes |
| Xian/2021 | Unclear | Unclear | Unclear | Yes | Unclear | Unclear | Unclear | Yes | Yes | Yes |
| Zhang M  /2022 | Unclear | Unclear | Unclear | Yes | Unclear | Unclear | Unclear | Yes | Yes | Yes |
| Zhang Y  /2022 | Unclear | Unclear | Unclear | Yes | Unclear | Unclear | Unclear | Yes | Yes | Yes |
| Yuan/2022 | Unclear | Unclear | Unclear | Yes | Unclear | Unclear | Unclear | Yes | Yes | Yes |

Yes, indicates low risk of bias; no, indicates high risk of bias; unclear, indicates an unclear risk of bias.
